# Supplementary figures and images for: The full transcription map of cottontail rabbit papillomavirus in tumor tissues
Source: PLoS Pathog. 2024 Oct 25;20(10):e1012649. doi: 10.1371/journal.ppat.1012649 (PMC11540226; doi:10.1371/journal.ppat.1012649)

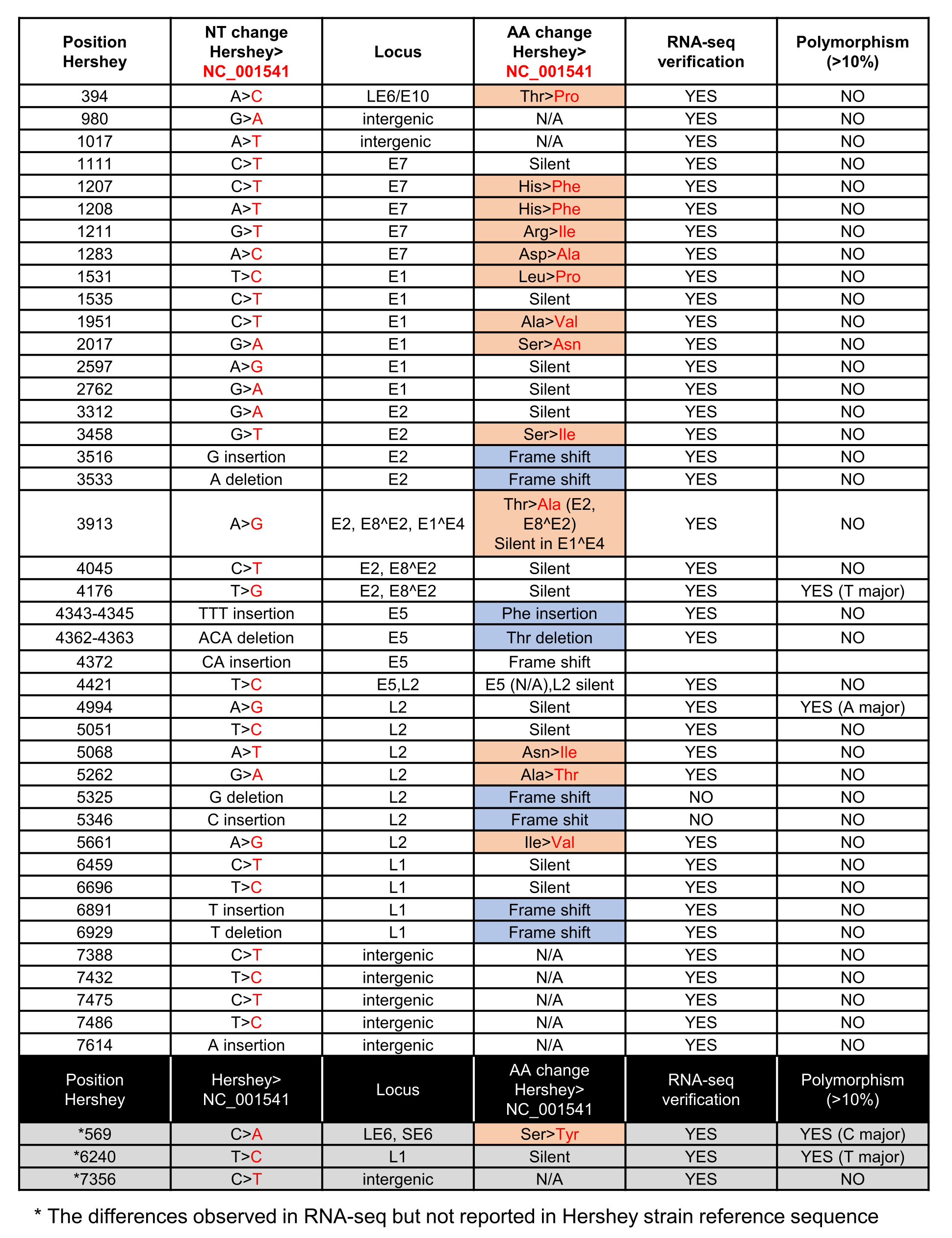

Supplement: S1 Fig — The nucleotide position, nucleotide change, locus, and type of mutations were detected by sequence alignment of the Hershey and Shope reference CRPV strains. (TIF) [file ppat.1012649.s001.tif]

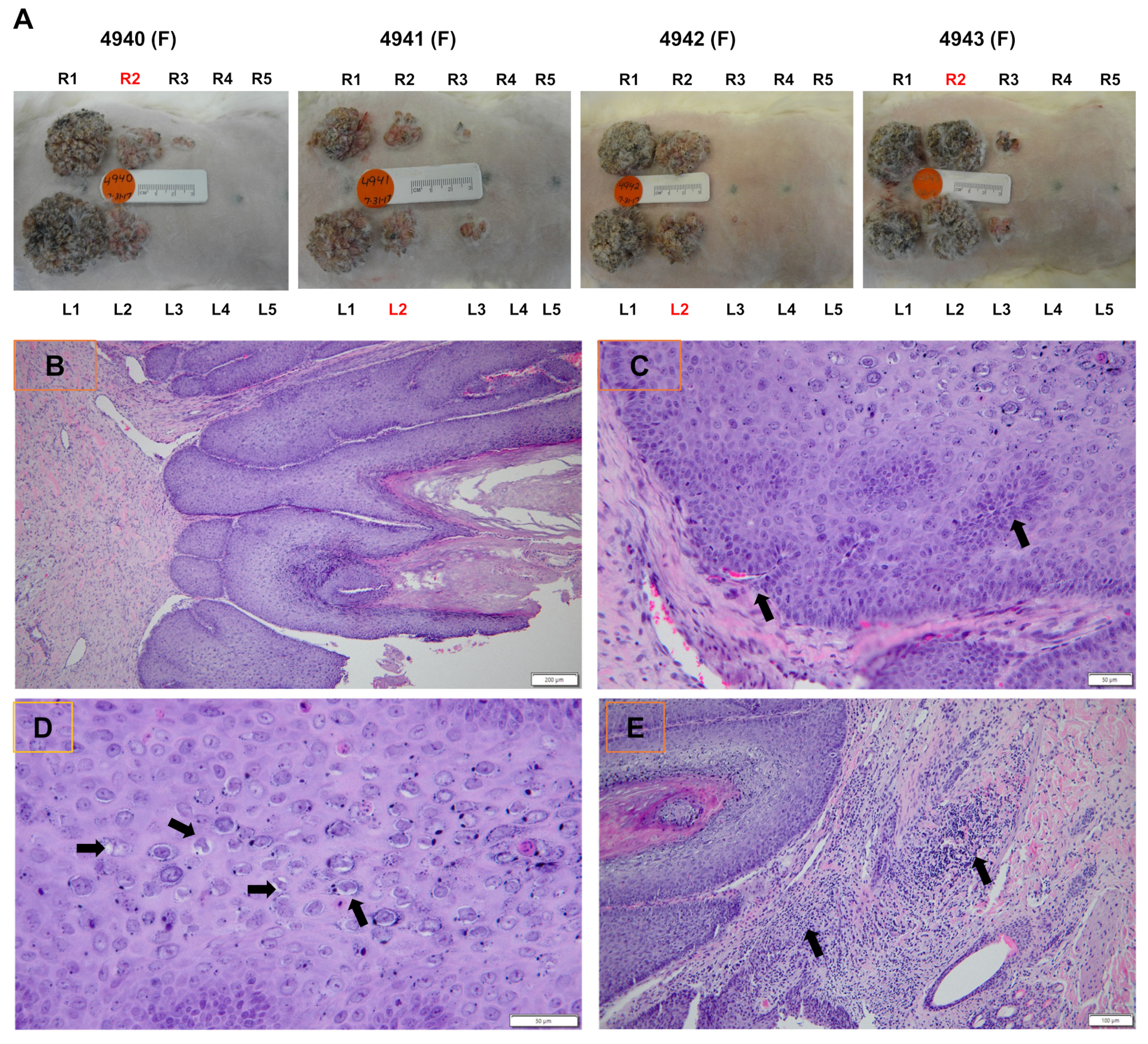

Supplement: S2 Fig — (A) The morphological appearance of CRPV-induced wart tissues on the back of four female (F) rabbits used in the study 8 weeks post inoculation with CRPV Hershey strain. R1-5 and L1-5 represent five sites inoculated with a 10-fold dilution of the virus inoculum on the right (R) or the left (L) side of the rabbit back. The wart tissues used for RNA extraction and RNA-seq are marked in red. (B-E) Histology of Hershey CRPV-induced wart tissues. On low magnification (B, 20×), these verrucous lesions form papillary projections of hyperplastic squamous epithelium covered by thick bands of lamellated keratin and variably supported by narrow fibrovascular cores. On higher magnifications (C, 40×), there is marked hyperkeratosis in addition to a severe expansion of the stratum basales with increased mitotic activity as well as thickening of the stratum spinosum (acanthosis) and stratum granulosum. Squamous epithelial cells in the superficial layers frequently display abnormally large nuclei surrounded by large, clear perinuclear halos (koilocytosis/koilocytotic atypia D, 60×, arrows). The superficial dermis is infiltrated by large numbers of lymphocytes in some samples (E, 40×, arrows). Histopathological features of malignancy were not identified. (TIF) [file ppat.1012649.s002.tif]

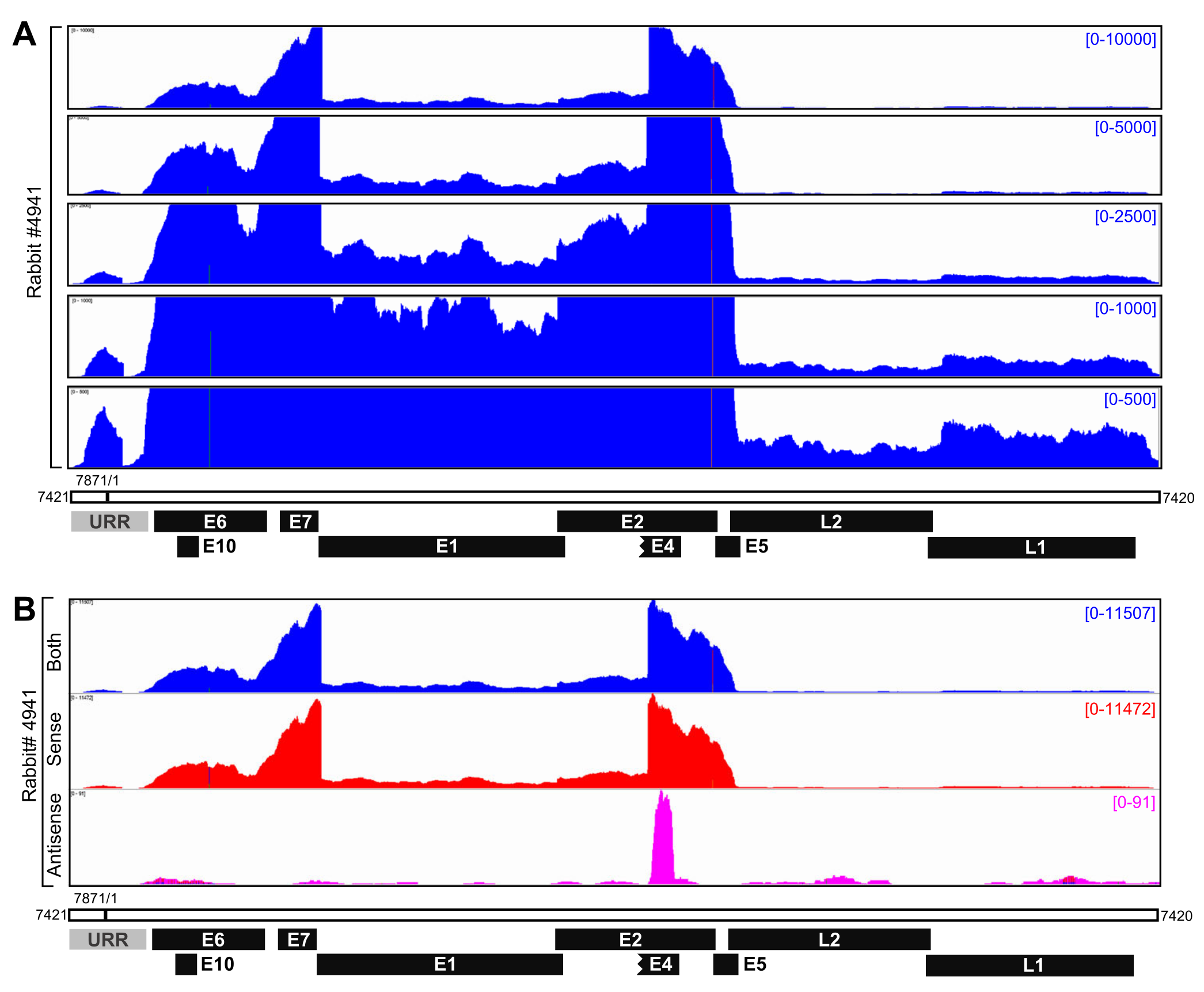

Supplement: S3 Fig — (A) The depth of RNA-seq reads coverage across the Hershey CRPV genome (GenBank Acc. No. JF303889.1) linearized at nt 7421 in one representative tissue sample (R4941) with a decreasing reads scale shown in the upper right corner. (B) The distribution of RNA-seq reads mapped to both (shown in blue), sense (shown in red), and antisense strand (shown in pink) of the CRPV Hershey genome visualized by IGV using the autoscale shown in the upper right corner. (TIF) [file ppat.1012649.s003.tif]

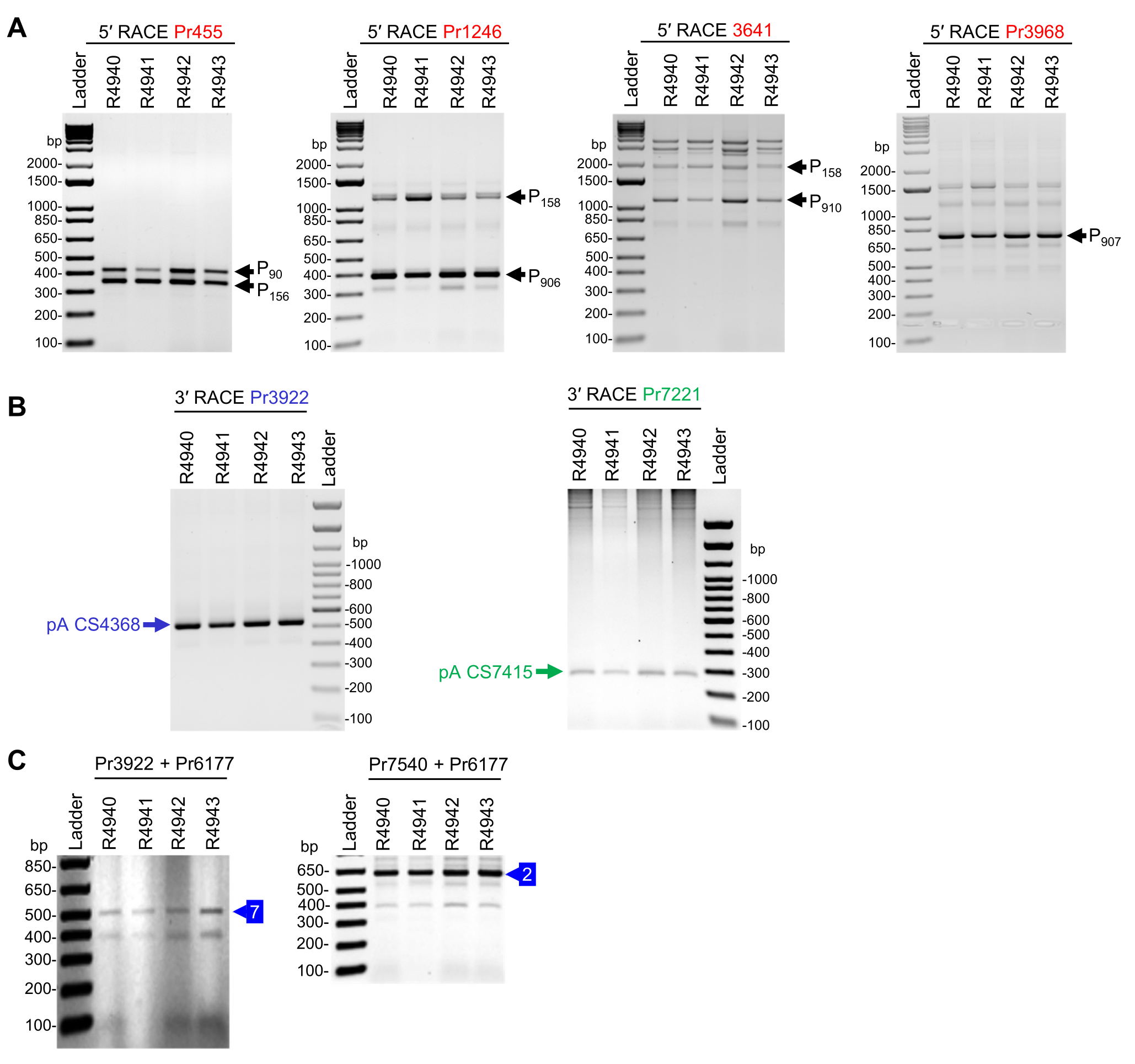

Supplement: S4 Fig — (A) The 5′ RACE for mapping transcription start sites. (B) The 3′ RACE for mapping RNA polyadenylation cleavage sites. (C) Validation by RT-PCR of newly identified splice sites for CRPV L1 expression. All assays were performed on cDNA generated from total RNA from four different animal tumor tissues used for RNA-seq. The primer sequences are in S7 Table. (TIF) [file ppat.1012649.s004.tif]

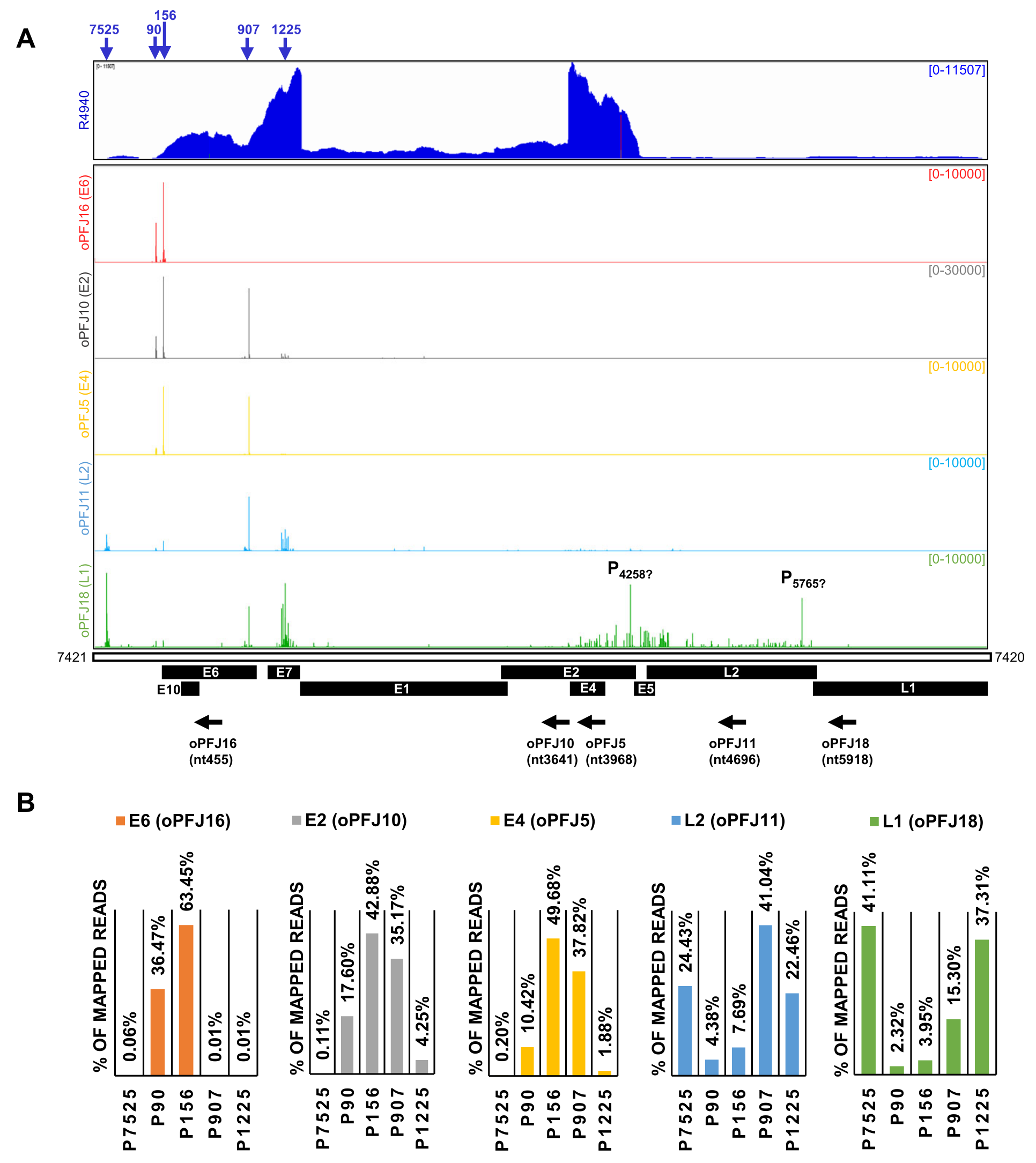

Supplement: S5 Fig — (A) The distribution of RNA-seq reads from one representative sample (R4941, upper panel) and viral transcriptional starting sites (TSS) determined by PacBio Iso-seq of 5′ RACE products obtained by primer antisense to various parts of the viral genome (black arrows beneath the diagram). The blue arrows at the top indicate the position of the major viral promoters (see Fig 2C). The black boxes below represent annotated viral ORFs. (B) The percentage of PacBio long reads from individual 5′ RACE libraries mapped to individual viral promoters (see S3 Table for more details). (TIF) [file ppat.1012649.s005.tif]

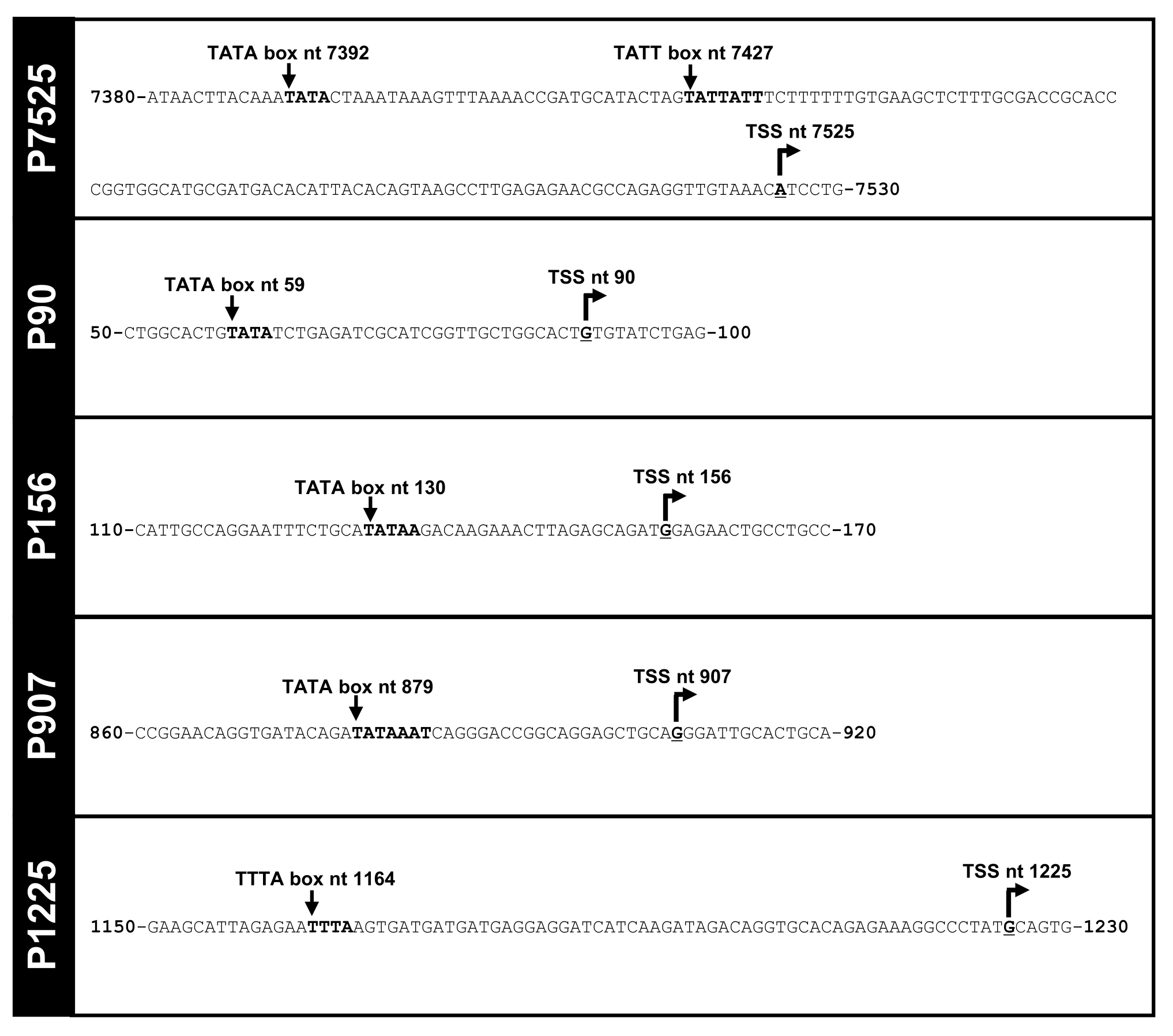

Supplement: S6 Fig — The nucleotide sequences of mapped CRPV promoter regions with predicted TATA or TATA-like box motifs positioned (indicated by arrows) upstream of the mapped transcriptional start site (TSS). (TIF) [file ppat.1012649.s006.tif]

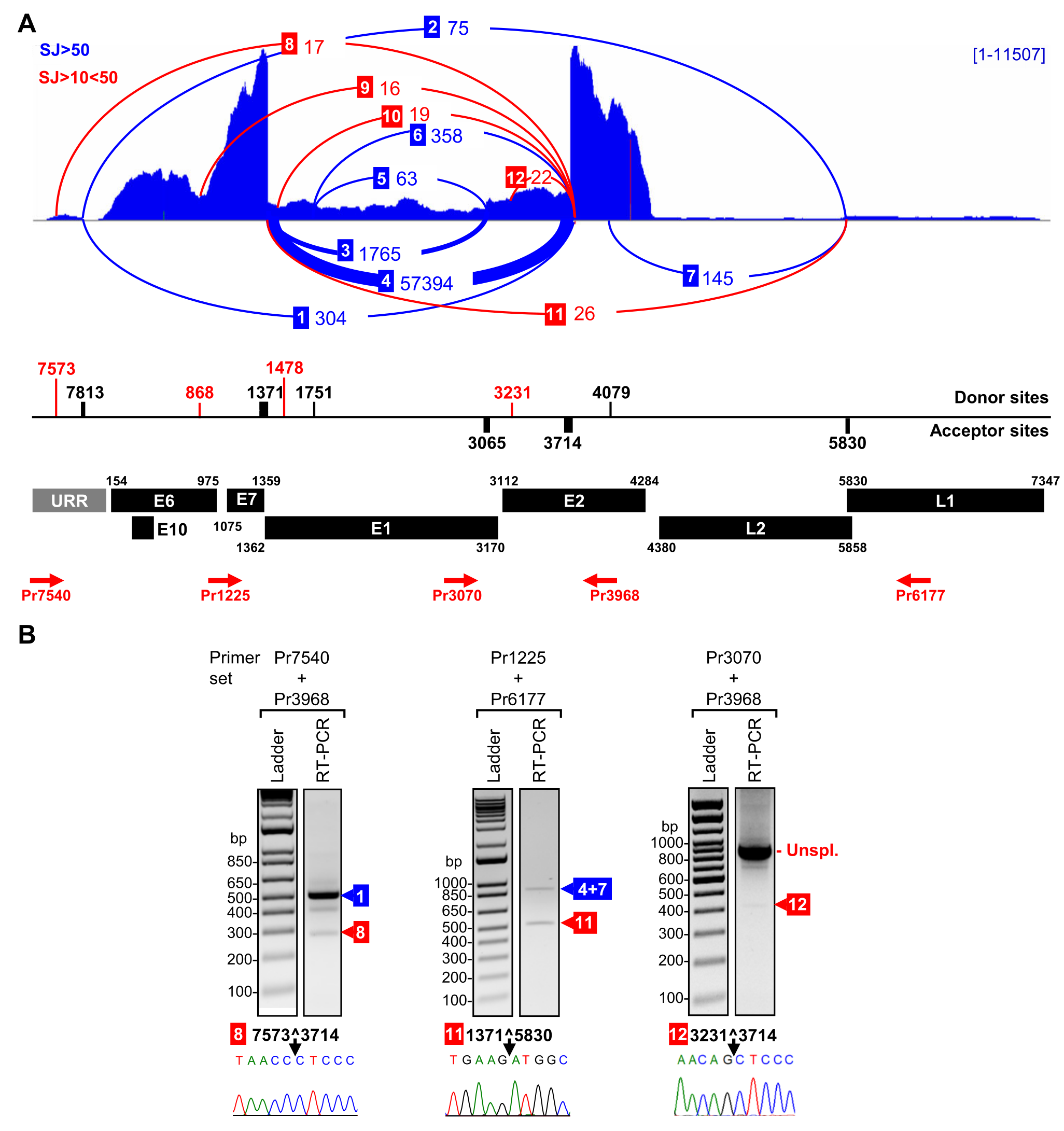

Supplement: S7 Fig — (A) RNA-seq coverage of the representative samples with a Sashimi plot of detected splice junctions (SJ) shown as the numbered arches (numbers 1–12 in the filled boxes). The associated number represents the number of total splice junction reads detected in all four samples (see S4 Table). The highly abundant SJ (>50 reads) are shown in blue. The additional less abundant SJ (>10<50 reads) are shown in red. The positions of minor splice sites labeled below the Sashimi plot are shown in red. The red arrows below the assigned viral ORFs (black boxes) represent the primers used in RT-PCR. (B) The gel electrograph of the RT-PCR product obtained using the primers shown in (A). The individual products were gel-purified and subjected to Sanger sequencing. The chromatographs with detected SJ are shown below. Unspl., unspliced product. (TIF) [file ppat.1012649.s007.tif]

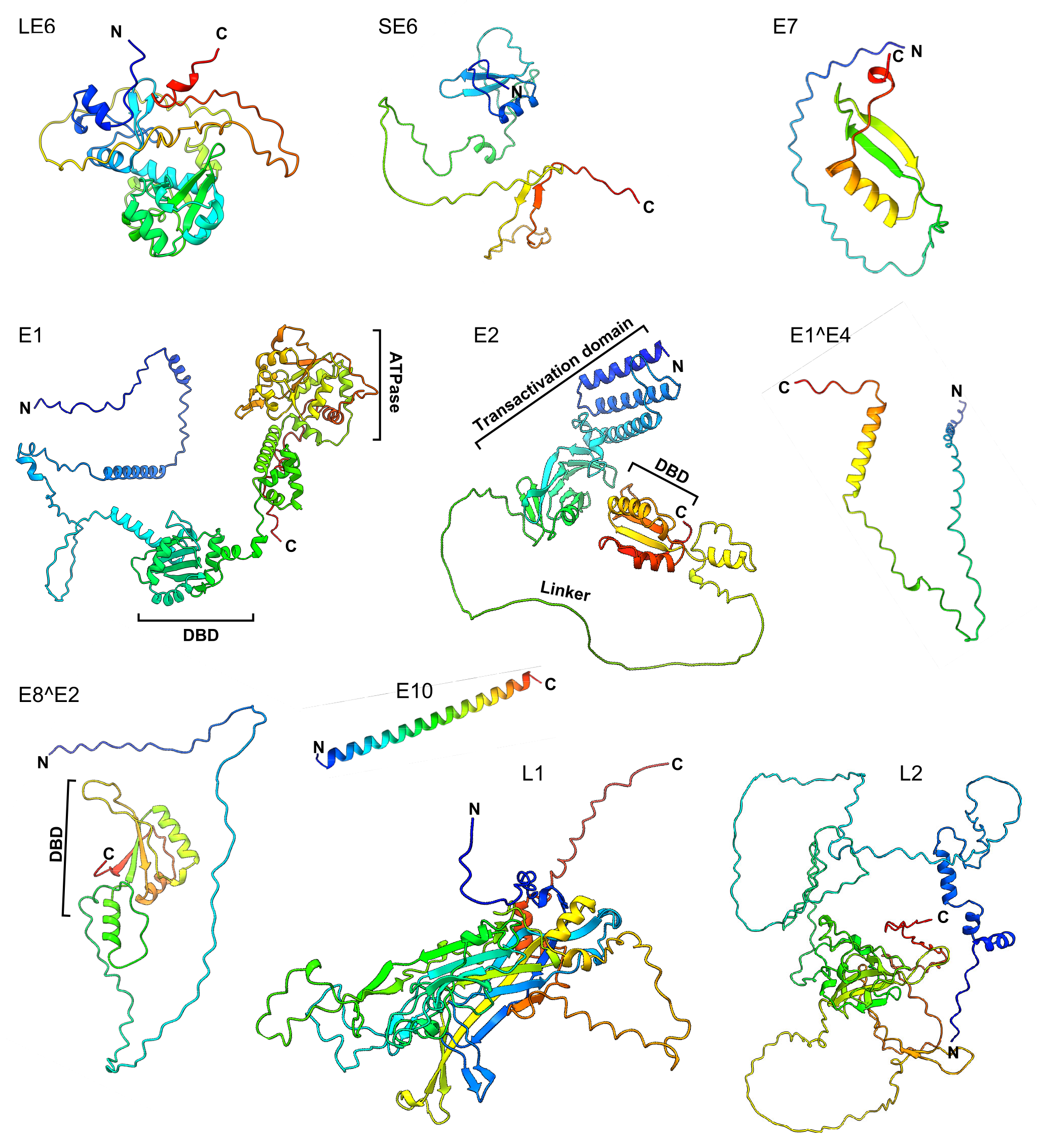

Supplement: S8 Fig — CRPV ORFs and their corresponding amino acid residues are shown in the S6 Table. The structure of each CRPV protein was predicted using the AlphaFold 2 algorithm with one representative model shown in the rainbow coloring with the N-terminus (N) marked as dark blue and the C-terminus (C) as dark red. The models shown are not in scale. See more details of the protein data bank (pdb) files in S1 Data for the individual protein structures predicted by AlphaFold 2. (TIF) [file ppat.1012649.s008.tif]

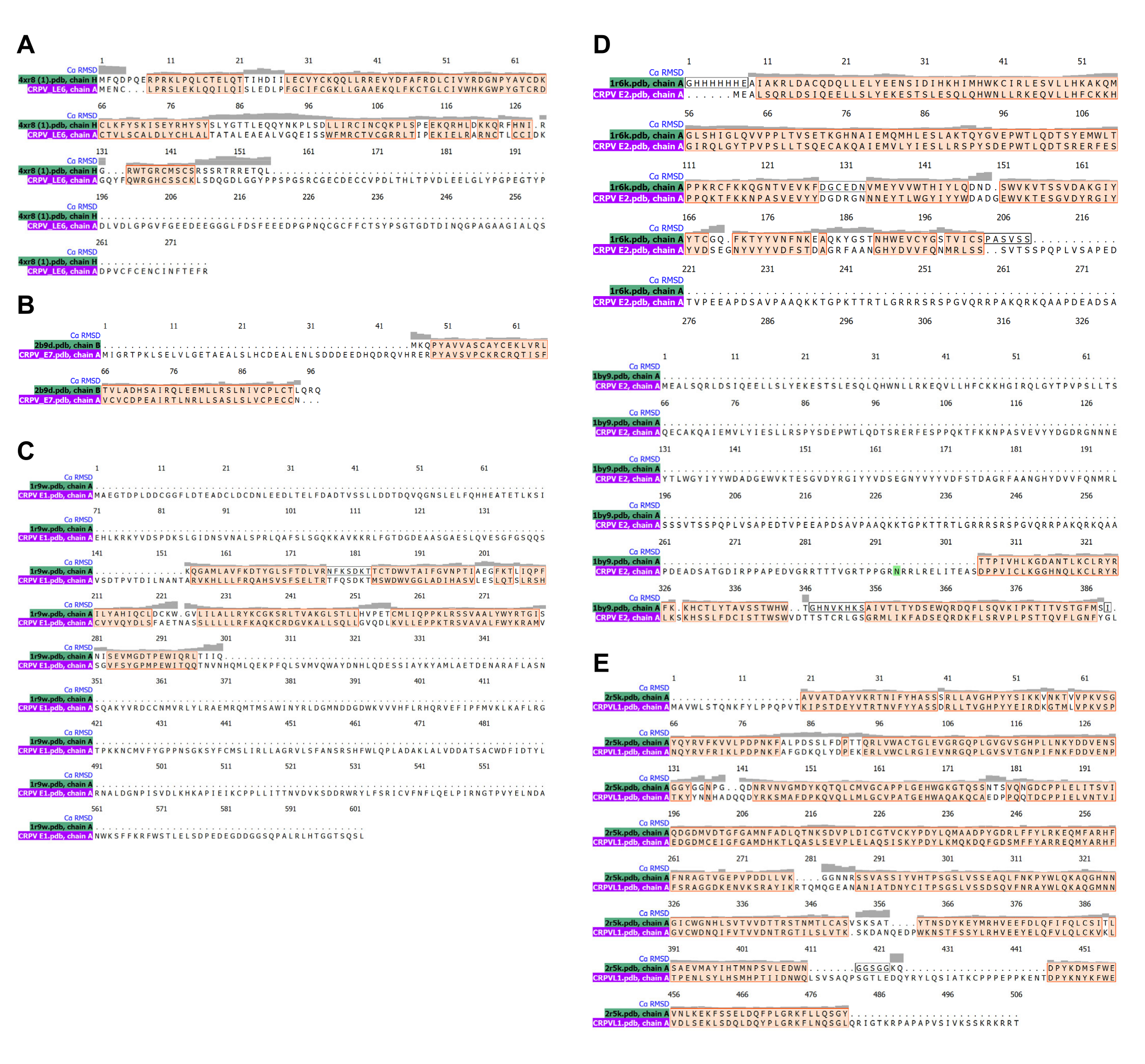

Supplement: S9 Fig — The matched residues are shown in yellow boxes. Ca RMSD in grey columns represents the single-point spatial variation among residues. All sequence alignments were performed by UCSF ChimeraX v1.8 using the Matchmaker showAligment function. (TIF) [file ppat.1012649.s009.tif]

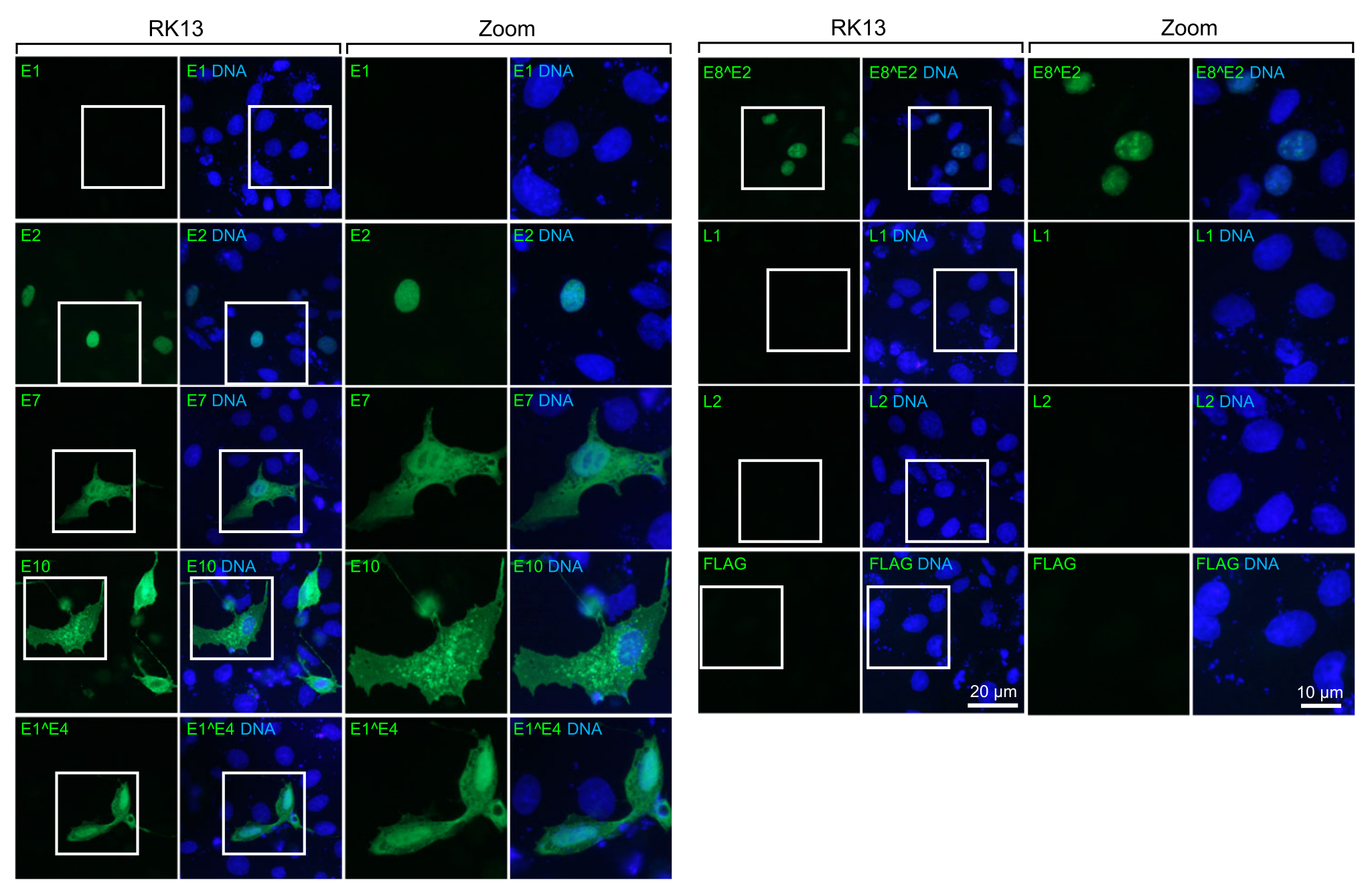

Supplement: S10 Fig — Rabbit kidney RK13 cells were transfected with the plasmids expressing FLAG-tagged CRPV proteins. Twenty-four hours after transfection, the cells were fixed and stained with an anti-FLAG antibody (green). The cell nuclei shown in blue were stained with Hoechst 33342 dye. (TIF) [file ppat.1012649.s010.tif]

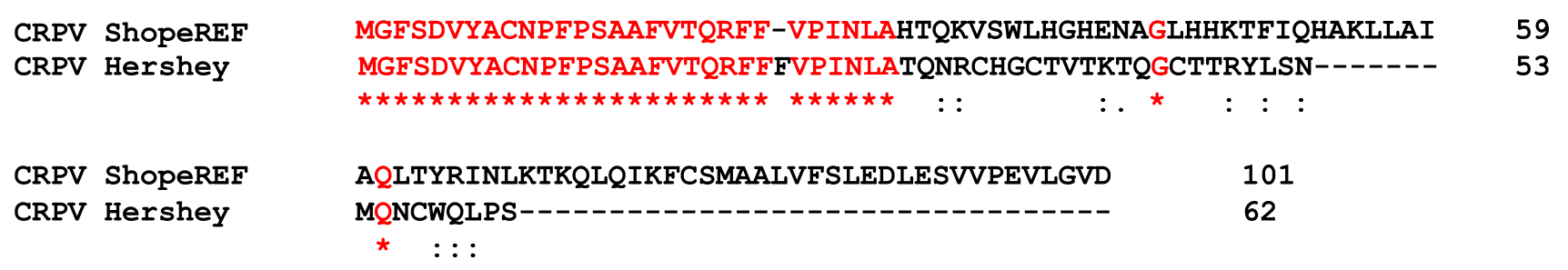

Supplement: S11 Fig — All sequence alignments were performed using the Clustal Omega software (www.ebi.ac.uk/Tools/msa/clustalo/). (TIF) [file ppat.1012649.s011.tif]
