# Supplementary material for: The full transcription map of cottontail rabbit papillomavirus in tumor tissues
Source: PLoS Pathog. 2024 Oct 25;20(10):e1012649. doi: 10.1371/journal.ppat.1012649 (PMC11540226; doi:10.1371/journal.ppat.1012649)
Supplement: Attachment — Submitted filename: Our rebuttals-8-28-2004.docx [file ppat.1012649.s020.docx]

**Our rebuttal responses to reviewer’s comments/questions**

**Part I - Summary**
Please use this section to discuss strengths/weaknesses of study, novelty/significance, general execution and scholarship.

Reviewer #1: Jiang et al. describe the complete transcript map of Cottontail rabbit papillomavirus using RNA derived from tumors induced in New Zealand White rabbits. They characterize transcription start sites, splice sites, and polyadenylation sites using a variety of standard mapping and state-of-the-art next generation sequencing methods. This results in an improved transcription map compared to the one provided on the PAVE web site. While this is impressive, the data mainly confirm findings (start sites, splice sites, differences of transcript levels) obtained in the past 35 years. The new findings are that CRPV also has a promoter in E7 driving late gene expression and its transcripts use a splice donor site in E4 and an acceptor site in front of L1. This study mainly confirms that human and animal PV have very predictable transcript patterns. The study lacks mechanistic insights and does not significantly enhance our understanding of PV. In summary, this a very well-executed study but it lacks novelty.

Thanks for your positive comments on our work, stating that our study “results in an improved transcription map compared to the one provided on the PAVE web site” and data are “impressive”, with new findings of promoter, new splice donor and acceptor sites….” from this very well-executed study. We believe our study provides a significant advancement for the field.

Reviewer #2: Analysing the transcriptome of cotton tail rabbit papillomavirus (CRPV) is important as it promises to add to our understanding of animal papillomaviruses. It will also allow comparison with human papillomaviruses, especially the cancer-causing ones. CRPV is an important model since it could be used as a in vivo model for testing therapies against human papillomaviruses and cancer progression. The manuscript contains for the first time a thorough analysis of the CRPV transcriptome. This information can be used in future to design new studies examining e.g. CRPV tumorigenesis. The results section follows the pattern of a previous transcriptomic study of the murine papillomavirus from this group. However, I believe the data could be curated better into far fewer figures, while the second part of the results in Figures 7, 9-11 is confusing in places and does not really add to the impact of this study.

Thanks for your positive comments, stating “The manuscript contains for the first time a thorough analysis of the CRPV transcriptome. This information can be used in future to design new studies examining e.g. CRPV tumorigenesis. As suggested, we have selectively revised our figures or added some new data to this revision.

Reviewer #3: In this study, the authors provide a comprehensive mapping study of the mRNA transcripts produced by the Cottontail Rabbit Papillomavirus. This now allows the position of the key viral promotors and polyA sites to be ascertained, and with the confirmation of splice donor and splice acceptor positions, it also allows the realisation of primary amino acid sequences. The authors have added tertiary structure predictions, and have also gone on to localise the expression patterns of some of the major mRNA species in CRPV-induced lesions using an RNA scope approach. In doing this, they have been careful to exclude signals derived from the possible amplification of viral episomes, which because of their abundance, can cause problems when trying to detect mRNA transcripts specifically in the upper epithelial layers. The expression studies in tissue culture provide a small amount of additional knowledge, with the main value being to provide data in support for previous work on CRPV protein function and tissue biology, and to pave the way for mor comprehensive studies on PV biology nd immunology in the future. This is a long overdue study, and the knowledge provided allowing a more substantial use of the CRPV system for basic science research and drug discovery in the future. The CRPV model is the best model of persistent infection in an immune competent host, and offers unique analysis opportunities when compared to the current mouse PV systems, which require immune deficient animals, and the rabbit oral papillomavirus system, which doesn’t support persistent infection. The Zheng group are world leaders in the analysis of PV RNA processing, and this study is of high quality.

Thank you for your very positive comments on our high-quality studies summarized in this report. Yes, a comprehensive CRPV transcriptome analysis is a long overdue study, and the knowledge provided allowing a more substantial use of the CRPV system for basic science research and drug discovery in the future. We thank our reviewer’s great appreciation that “the Zheng group are world leaders in the analysis of PV RNA processing, and this study is of high quality”.

**Part II – Major Issues: Key Experiments Required for Acceptance**
Please use this section to detail the key new experiments or modifications of existing experiments that should be absolutely required to validate study conclusions.

Generally, there should be no more than 3 such required experiments or major modifications for a "Major Revision" recommendation. If more than 3 experiments are necessary to validate the study conclusions, then you are encouraged to recommend "Reject".

Reviewer #1: (No Response)

Reviewer #2: While it is clear that RNASeq was performed on tumors from 4 x male and 4 x female rabbits, it is not clear from the materials and methods section, or the figure legends, if the subsequent 5’-, 3’-RACE and RT-PCR data were carried out on more than one tumor tissue. This is important since individual animals are being investigate and there will be inter-tumor and inter-individual variation. For the RT-PCR data, amplification of an internal control is required since relative band intensities are discussed.

This is an excellent comment. Yes, we have analyzed more than one tumor tissue including tumor tissues from other animals and concluded the same results on mapped promoters, splice sites and pA site usages. Data are now summarized in a supplemental Fig S4.

The transcriptomic data are clear and carefully documented but I suggest that the data could be summarized better. e.g. in Figure 1, if the transcriptome profile for the viral genomes from each rabbit was very similar, why not just show Fig1D and add a table to show the % reads for each of the four replicates.

Thanks for your suggestions. We would like to show that all CRPV transcriptome profiles are similar visually from individual rabbits to impress our readers. As the reviewer pointed out above, it is important to show our data were carried out on more than one animal tissues with the consistent profile. Fig 1D is to show strand-specific CRPV RNA transcription, indicating that CRPV, as seen from other papillomaviruses, transcribes only from its minus DNA strand as a template DNA and thus viral RNA is in sense orientation. We barely detect viral RNA in antisense orientation.

There are several repetitions of the viral genome map (Figs 2, 3, 4, 5, 7,8) and the transcriptome of rabbit #4940 (Figs 1,2 & 3). This detail is not required in every figure. For figures where primers or probes are used, tables giving the locations and sequences of the various primers used would be helpful. Naming the primers/probes in the various panels of all the figures e.g. E6 probe1 or E1^E4 primer pairs X etc. would then allow the reader to refer to the primer/probe table.

Thanks for your suggestions. We have combined Fig 4 and Fig 5 as one Fig 4. Viral genome map in Fig 2 and Fig 3 are different. The latter has the mapped promoters from Fig 2 on the linearized genome in correlation to the transcriptome profile, which is important for our readers to know viral RNA in polycistronic nature and subjects to RNA splicing and polyadenylation regulations. All primer sequences and locations are summarized in Table S7. Sorry, we can’t label our probe as an E6 probe or an E4 probe in Fig. 7 and Fig. 8 in our previous submission (now Fig. 6 and Fig. 7 in this revision) because this label will mislead the field and data interpretations. A probe positioning on an E6 or E4 ORF region is not specific for E6 or E4 detection because of the polycistronic nature of viral RNA transcripts.

Figure 7 is unhelpful due to the poor quality of the northern blots. The authors cannot conclude from the data what transcript each band represents, whether bands represent one or multiple transcripts or whether there are the same or different bands between panels. The northern blot data do not add to the transcriptomic and RT-PCR data and should be removed.

We appreciated the reviewer’s challenge on Northern blot quality in Fig. 7 (now Fig. 6 in this revision). In this revision, we have replaced these blots with high quality of images. We disagree with this reviewer about the identity of the Northern blot bands, which we labeled clearly what band represent which transcripts by calculation (S5 Table). We also believe that our Northern blot data are consistent with our transcription profile and RT-PCR data and provide more weights as additional validations. As shown in Fig. 5, the reviewer may also notice that all viral early transcripts, no matter which promoter is used for transcription, are polyadenylated at early pA site and thus contain multiple ORFs and are polycistronic. The concept on one gene and one RNA to make one protein does not apply to any papillomavirus. As such, we would like to remain the Fig. 6 in this revision in hope that our reviewer agrees with our decision.

The Alphafold predictions in Figure 9 can only be useful if modelled on top of the corresponding viral proteins from other PVs as has been done for CRPVLE6 and HPV16 E6. On top of this, considerably more annotation would be required, together with 3D images. I don’t believe these data, as they stand, add to the manuscript and suggest they be removed.

Again, thanks for the suggestion. We have modified the Fig. 8 (Fig. 9 in the previous submission) by modelling to the corresponding papillomaviral protein structures available in the literature. The reviewer probably knows that only a few viral proteins in papillomavirus field are available. Thus, we have other CRPV proteins in the Alphafold predictions now in the supplemental figure Fig S8. The structure alignments of their counterparts are in Fig S9, with .pdb files for the HPV proteins comparable to the corresponding CRPV proteins.

The western blots in Figure 10 cannot be considered quantitative unless loading controls and replicates are performed and relative quantification of protein expression is carried out. For example, although the overall protein levels are much less in RK13 cells compared to HEK293 cells, it looks like there is at least as much increase in LE6 by inhibiting the proteasome in RK13 cells as in HEK293 cells. In addition, in Figure 10C, I don’t understand the relevance of comparative expression of FLAG-tagged CRPV proteins in the two cell lines especially as there is no internal loading control.

Thanks for your critical comments. The loading control (tubulin) for each panel in Fig. 9 (Fig. 10 in our previous submission) has been added in this revision. Although these proteins have been studied individually for their functions, this study is first time for comparison of all CRPV proteins side-by-side for their expression capability under a strong heterologous CMV IE promoter in RK13 and HEK293T cells (new Fig 9 and S10 Fig). We demonstrated that E2, E10, E1^E4, E8^E2, but not others, could be easily expressed in both cell lines. LE6 can be easily expressed also in HEK293T cells but not in RK13 cells. Interestingly, we found LE6 expression in both cell lines could be increased in the presence of MG132, a proteasome inhibitor, which shows such a function for SE6 only in HEK293T cells. One of three immunoblots was shown as a representative of three experiments.

In Figure 11, rabbit and kidney cell lines are used to try to determine the location of expression of the CRPV proteins in cells. This experiment is flawed since it does not take account of the normal host cell of the virus, which is keratinocytes. More importantly, PV gene expression responds to epithelial differentiation. It should be possible to carry out raft culture of primary or immortalized rabbit keratinocytes to address the cell type issue and to properly assess the subcellular location of viral proteins in a tissue, especially the late proteins since they are expressed in differentiated keratinocytes.

As we stated above, 10 CRPV proteins have not been compared side-by-side for their expression capability by transient overexpression commonly performed for functional studies of individual proteins. Thus, we carried out such study in RK13 and HEK293T cells and have each CRPV gene (ORF) under a strong heterologous CMV IE promoter control for transcription, of which promoter activity does not depend on epithelial differentiation. As a continuing effort, we determine the localization of expressed CRPV proteins in these two types of cells (new Fig 10, Fig S10) and concluded that in both types of cells, viral LE6, SE6, E1, E2, E8^E2 could be easily expressed as a nuclear protein, E7 and E1^E4 expressed both in the nucleus (more for E7) and cytoplasm (more for E1^E4), and E10 expressed primarily as a cytoplasmic protein. However, L1 could be expressed as a nuclear protein only in HEK293, but not expressible in RK13 cells and L2 is not expressible in both types of cells (new Fig. 10, Fig. S10 in this revision). We think these data will be useful for future studies.

Reviewer #3: None

**Part III – Minor Issues: Editorial and Data Presentation Modifications**
Please use this section for editorial suggestions as well as relatively minor modifications of existing data that would enhance clarity.

Reviewer #1: l. 103-106: More recent studies investigating interactors of E6 should be also described and included (PMID: 20663910; PMID: 18067942, PMID: 36155393)

Thanks. The suggested references are cited in this revision (lines 109-110).
l.119: it would be more appropriate to mention/reference the PAVE web site for HPV16 and 31 (and other PV) instead of ref. 67 and 68. Ref. 70 has nothing to do with PV transcript maps and needs to be removed.

PAVE website has been added and Ref. 70 has been deleted in this revision. Ref. 67 and 68 remain without deletion for the original and updated transcription map information (Line 128).

l. 211/212: the late promoter in E7 was first identified in HPV31 and 16 by the Laimins lab (PMID: 1326657) and the Iftner lab (PMID: 8642661). These references should be included.

Thanks. Cited accordingly (Lines 223-224).
l. 227: heterogenous start sites for the HPV31 late promoter were first described in PMID: 9525589. Please add.

Cited in this revision (Lines 238-239).
l. 347-349: CRPV E5 is present on L2 transcripts and thus might be a late protein. I therefore recommend to include it on the map and modify the sentence accordingly.

Thanks for the suggestion for E5 inclusion. However, the putative CRPV E5 ORF is positioned at nt 4274 and 4462 in the CRPV Hershey genome and the mapped early RNA polyadenylation cleavage site is mapped to the genome nt 4368. Thus, the early transcripts most likely do not have an intact E5 ORF. Yes, the E5 might present on L2 transcripts, but L2 translates from a different frame starting within the E5 ORF and bears a strong Kozak sequence (….RccAUGG….), with a purine both in -3 and +4 positions (Kozak M. NAR 15: 8125–8148, 1987). In contrary, E5 ORF does not have a strong Kozak sequence, but a ….tttttAUGG….. sequence. This feature of E5 on L2 transcript will not make E5 translatable, but would prefer L2 translation. Moreover, in vivo studies demonstrated that the E5 ORF in the CRPV genome is dispensable in tumor induction (PMID: 1326666) and E5 DNA vaccination failed to provide protection against viral challenge (PMID: 17603848). Accordingly, we would like our reviewer to agree with us to remain the statement that E5 is inexpressible in the context of CRPV genome (Lines 365-367; Lines 542-565).

CRPV E1, E2, L/SE6, E7, E8^E2, and E10 (E8) have been functionally investigated by several groups. Simple overexpression of these proteins and analysis by immunoblot and immunofluorescence does not really add new information compared to previous work. Therefore, Fig. 9 – 11 and l.433-484 can be deleted from the results section.

Although these proteins have been studied individually for their function, they have not been compared for all CRPV proteins side-by-side of their expression capability even under a strong heterologous CMV IE promoter in RK13 and HEK293T cells. We demonstrated that E2, E10, E1^E4, E8^E2, but not others, could be easily expressed in both cell lines (new Fig. 9 and Fig. 10, Fig. S10 in this revision). LE6 can be easily expressed also in HEK293T cells but not in RK13 cells (new Fig. 9). Interestingly, we found LE6 expression in both cell lines could be increased in the presence of MG132, a proteasome inhibitor, which shows such a function for SE6 only in HEK293T cells (new Fig. 9).

A new Fig. 8 revised from the Fig. 9 in our previous submission has provided by modelling CRPV proteins to the corresponding papillomaviral protein structures available in the literature. All other CRPV proteins in AlphaFold2 prediction are now moved to the supplement Fig S8.

I miss a statement and references in the results and/or discussion section that four CRPV promoters and five splice sites have been discovered before by other groups.

All four CRPV promoters are acknowledged in the results in our previous submission (Lines 218-222) with reference citations. A partial transcription map of CRPV early region also mentioned in our previous submission (Line 120) with citations. To be more specific, we added one sentence in this revision that five splice sites, 7813^3714, 1371^3065, 1371^3714, 1751^3065, 1751^3714 were reported by other labs with references (Lines 286-287).

Reviewer #2: Why does LE6 staining not go way in the presence of MG132 in Figure 11, as suggested by Fig 10? There is also a lot of nuclear staining for E1^E4 in this figure despite what the authors claim.

As MG132 is a proteasome inhibitor and LE6 is subject to proteasome degradation (new Fig. 9 in this revision), LE6 level should be increased in the presence of MG132, but not go away. Recent studies suggest that E1^E4 might have nuclear functions (PMID: 15890911) and the E1^E4 proteins of the Beta, Gamma and Mu HPV types sometimes assemble into distinctive nuclear inclusion granules (PMID: 24016539).

The discussion could focus on comparison with other PVs and mention cancer progression.

As this report is a transcription mapping study, not a CRPV oncogenesis study, our discussion has focused on CRPV transcription and post-transcription issues and possible variations from a variable CRPV genome due to mutation or deletion. Many comparisons with other PVs have been made in results.

On lines 423 and 432 “corneal layers” is incorrect, they mean “cornified” layers.

Corrected in this revision. Thanks.

Reviewer #3: 1. Previous work has indicated that the Cottontail Rabbit Papillomavirus is not able to efficiently complete its productive life cycle in domestic laboratory rabbits, but in this study, the authors show evidence of productive genome amplification in the New Zealand White rabbit background. Could the authors add some additional dialogue to help the reader to understand this.

Thanks for your comments. Yes, we have published L1 protein staining (when using a better antibody G4B than the old anti-GSA antisera) (Figure below) and retrieved some residual infectious virions from lesions (PMID: 18024897). We also detected serum conversion to L1 in infected rabbits (PMID: 18024897). CRPV L1 expressing plasmids generated protective T-cell responses to viral DNA challenge (PMID: 16987067). We would agree that virion production is less than what is found in the natural host (cottontail rabbits). Others have located late transcripts in domestic rabbit lesions (PMID: 8189500, PMID: 26297146). Thus, a productive genome amplification and late transcript expression in domestic laboratory rabbits found in this report are not surprising.

Published Fig 2b by JGV 8: 3286-93, 2007. Improved L1 staining in rabbit tissues by an anti-CRPV L1 antibody G4B (lower panel) compared to GSA (upper panel) (PMID: 18024897).

2. It is not always clear which transcripts are ‘major’ transcripts, and which cases are minor species. This is important when considering protein function, particularly for transcripts that map to E2, which is a transcription factor that occurs as forms that drive transcriptional activation and repression of the viral early promotors. If the authors could add some additional commentary, this would be helpful.

We have labeled in Fig. 5 for relative abundant transcripts in red color. E2 transcripts should be minimal as shown in Fig. 4A because of RNA splicing and E2 translation is most likely from the P156 and P907-derived transcript L and T (Fig. 5). We have added this description in Lines 341-342).

PLOS authors have the option to publish the peer review history of their article ([what does this mean?](https://gcc02.safelinks.protection.outlook.com/?url=https%3A%2F%2Fjournals.plos.org%2Fplospathogens%2Fs%2Feditorial-and-peer-review-process%23loc-peer-review-history&data=05%7C02%7Czhengt%40exchange.nih.gov%7C05c0056d7492428e7c5c08dc99d0571d%7C14b77578977342d58507251ca2dc2b06%7C0%7C0%7C638554367313734296%7CUnknown%7CTWFpbGZsb3d8eyJWIjoiMC4wLjAwMDAiLCJQIjoiV2luMzIiLCJBTiI6Ik1haWwiLCJXVCI6Mn0%3D%7C0%7C%7C%7C&sdata=YlJD7nAcs0y38U1767j1IaolW7U0HvCtNt76%2F7c%2BwPA%3D&reserved=0)). If published, this will include your full peer review and any attached files.


If you choose “no”, your identity will remain anonymous but your review may still be made public.


**Do you want your identity to be public for this peer review?** For information about this choice, including consent withdrawal, please see our [Privacy Policy](https://gcc02.safelinks.protection.outlook.com/?url=https%3A%2F%2Fwww.plos.org%2Fprivacy-policy&data=05%7C02%7Czhengt%40exchange.nih.gov%7C05c0056d7492428e7c5c08dc99d0571d%7C14b77578977342d58507251ca2dc2b06%7C0%7C0%7C638554367313744738%7CUnknown%7CTWFpbGZsb3d8eyJWIjoiMC4wLjAwMDAiLCJQIjoiV2luMzIiLCJBTiI6Ik1haWwiLCJXVCI6Mn0%3D%7C0%7C%7C%7C&sdata=a90YHfYKwPXn5asZC6ECWZV%2FVClep4zELhW9BAr2EAo%3D&reserved=0).

Reviewer #1: No

Reviewer #2: No

Reviewer #3: No
