# Supplementary material for: The full transcription map of cottontail rabbit papillomavirus in tumor tissues
Source: PLoS Pathog. 2024 Oct 25;20(10):e1012649. doi: 10.1371/journal.ppat.1012649 (PMC11540226; doi:10.1371/journal.ppat.1012649)
Supplement: Attachment — Submitted filename: Our responses to reviewer#2.docx [file ppat.1012649.s021.docx]

Our responses to reviewer #2’s minor questions

**Part III – Minor Issues: Editorial and Data Presentation Modifications**
Please use this section for editorial suggestions as well as relatively minor modifications of existing data that would enhance clarity.

Reviewer #2: The authors have revised their manuscript according to the reviewers’ comments. The work is now more robust and more easily understood in relation to work on other papillomaviruses. The new alphafold figure is particularly helpful.

I still have concerns regarding the northern blots. The images are improved but, in general, the resolution of northern blots does not allow multiple transcripts of similar sizes to be definitively assigned to bands. For example, the description of Blot 1 does not correspond to the text “As shown in Fig 6B, Fig 5C, and S5 Table, Probe 120 immediately downstream of P90 detected three distinct products, the most abundant ~1.9-kb transcript D with single nt 1371^3714 splicing and less ~2.2-kb transcript G with nt 1751^3714 splicing, ~2.5-kb transcript E with nt 1371^3065 splicing,….”

The text states that three products are seen but goes on to describe four mRNAs. The most abundant transcript looks to be greater than 2kb, not 1.9 kb as stated. Is it possible to be certain that there are two bands around 2 kb considering the resolution in this part of the gel? The band representing transcript E at ~2.5 kb cannot be seen in this blot.

It seems that the authors are assuming that the bands in the blots, e.g. the major band at ~2 kb, represent multiple transcripts but due to the resolution of the gels, it should be made clear in the text that the bands were assigned by presumption from the transcriptomic and RT-PCR data.

Thanks for the constructive comments. We have revised our Fig. 6B and deleted the 2.5-kb transcript E from the probe 120 blot and E, L, X transcripts from the probe 972 blot because they are barely seen on the blot. We agree with the reviewer about our bands assigned based on our transcriptomic and RT-PCR data. Accordingly, we revised our statement in the revised manuscript (see lines 403-404).
